# The role of sleep deprivation and fatigue in the perception of task difficulty and use of heuristics

**DOI:** 10.5935/1984-0063.20180016

**Published:** 2018

**Authors:** Mindy Engle-Friedman, Gina Marie Mathew, Anastasia Martinova, Forrest Armstrong, Viktoriya Konstantinov

**Affiliations:** 1 Baruch College, City University of New York, Psychology - New York - NY - USA.

**Keywords:** Sleep Deprivation, Fatigue, Perception, Heuristics, Effort-Mental, Decision Making, Motivation

## Abstract

This study investigated the effects of sleep deprivation on perception of task difficulty and use of heuristics (mental shortcuts) compared to naturally-experienced sleep at home. **Methods:** Undergraduate students were screened and assigned through block-random assignment to Naturally-Experienced Sleep (NES; *n*=19) or Total Sleep Deprivation (TSD; *n*=20). The next morning, reported fatigue, perception of task difficulty, and use of “what-is-beautiful-is-good,” “greedy algorithm,” and “speed-accuracy trade-off” heuristics were assessed. **Results:** NES slept for an average of 354.74 minutes (*SD*=72.84), or 5.91 hours. TSD rated a reading task as significantly more difficult and requiring more time than NES. TSD was significantly more likely to use the greedy algorithm heuristic by skipping instructions and the what-is-beautiful-is-good heuristic by rating an unattractive consumer item with a favorable review as poor quality. Those in Total Sleep Deprivation who chose more difficult math problems made this selection to finish the task more quickly in findings approaching significance, indicating use of the speed-accuracy trade-off heuristic. Collapsed across conditions, self-reported fatigue predicted greater perceived difficulty in both the reading task and a visuo-motor task, higher quality rating for the attractive consumer item, and lower quality rating for the unattractive consumer item. **Conclusions:** Findings indicate sleep deprivation and fatigue increase perceptions of task difficulty, promote skipping instructions, and impair systematic evaluation of unappealing stimuli compared to naturally-experienced sleep.

## INTRODUCTION

Elevated fatigue in critical decision making is associated with costly real-world outcomes. In a sample of 204 primary care physicians, the prescribing of antibiotics meeting the “sometimes indicated” and “never indicated” criteria increased progressively over three-hour work sessions^1^. A study of 4,000 health care workers found an 8% decline in the frequency of hand washing over the course of the work shift^2^. In a study of parole verdicts, judges made progressively fewer favorable verdicts (which are more demanding than unfavorable verdicts) from 65% favorable at the start of the session to 0% favorable at the end^3^. Fatigued individuals respond with racial bias by producing shorter reaction times in a shooting simulation of Black armed suspects, depictions which support stereotypes^4^.

Fatigue, the subjective feeling of tiredness and exhaustion, accompanies experimentally controlled sleep deprivation^5^^,^^6^, naturally occurring poor quality sleep^7^, and insufficient sleep^8^, and seems to be associated with behavioral reductions in effort. Though loss of sleep typically leads to greater fatigue, there is considerable variability in subjective feelings of fatigue following sleep loss^9^. Total sleep deprivation in the experimental setting refers to twenty-four hours or more of extended wakefulness not induced by naturalistic causes, such as illness. Such experimenter-controlled total sleep deprivation results in objective increases in simpler behaviors, including fewer attempts at problems^10^, the choice of low-effort/low-monetary reward tasks over high-effort/high-reward tasks^11^, the selection of easier math problems^12^, and increased number of unsolved math sequences when informed of task difficulty^13^.

Sensations that accompany sleep deprivation, such as fatigue, may reflect physical or cognitive limitations and signal reductions in capacity that directly affect behavior. Reductions in task engagement may result from a heightened perception of task difficulty caused by the absence of sleep and the fatigue that ensues. For example, fatigued individuals evaluated a hill as steeper and physical distances as greater^14^, though participants did not actually measure hill steepness or physical distance.

In a sample of ice skaters, reports of less sleep and more frequent awakenings were associated with perception of greater difficulty of certain skating maneuvers, and poorer sleep quality predicted choice of easier maneuvers^15^. In studies of college students, math tasks perceived to be less difficult were chosen following sleep deprivation compared to those chosen following full sleep^12^. As perception precedes behavior^16^, increased perceptions of task difficulty could help explain alterations in behavior following sleep deprivation.

Heuristics, as defined by Simon^17^, are cognitive strategies in decision making which are used to obtain adequate solutions while minimizing systematic processing. Heuristics involve simpler strategies to arrive at solutions, such as examining fewer cues or integrating less information^18^. Thus, decisions made through heuristic processing rely on less effortful strategies than those made through systematic processing^19^. In the one total sleep deprivation study that assessed heuristics, an increased use of the local-representativeness heuristic was observed; participants were unable to suppress pre-potent biases following sleep deprivation^20^. An understanding of the specific heuristics used following sleep deprivation in the laboratory can help to identify similar applied situations in which heuristics may be used.

The present study examined the impact of sleep deprivation on the use of three types of heuristics: the “what-is-beautiful-is-good” heuristic^21^ in which a stimulus perceived to be physically attractive (“what-is-beautiful”) is judged to be inherently more valuable (“good”) than an unattractive stimulus, the “greedy algorithm” heuristic^22^, in which minimal time is used to reach a solution rather than a systematic evaluation of information, and the “speed-accuracy trade-off”^23^, in which effort is conserved to end task engagement.

Use of these heuristics can negatively impact productivity and quality of life. Using the what-is-beautiful-is-good heuristic can lead to discrimination in the workplace; physically attractive individuals receive higher starting salaries^24^ and better performance evaluations^25^ than less attractive individuals. Use of the greedy algorithm heuristic may result in the worst possible solution^26^, and by choosing the more expedient option with the speed-accuracy trade-off, tasks completed more quickly may contain more errors^27^.

The aim of this study was to determine whether total sleep deprivation produces greater perception of task difficulty and use of heuristics compared to naturally-experienced sleep. We hypothesized that in comparison to those with naturally-experienced sleep at home, sleep-deprived participants would (1) rate certain tasks as more difficult, (2) perceive greater task difficulty of task-specific elements, and (3) use the what-is-beautiful-is-good, greedy algorithm, and speed-accuracy trade-off heuristics rather than complex mental processes. We further hypothesized that (4) increased levels of reported fatigue would predict greater perception of difficulty and heuristic use, regardless of experimental condition.

## METHODS

### Participants

Thirty-nine undergraduate students (17 female) from the ages of 18 to 29 (*M*=19.18, *SD*=2.67) were assigned through block-random assignment to Naturally-Experienced Sleep (NES; *n*=19) or Total Sleep Deprivation (TSD; *n*=20). The sample was 49% Asian (19), 18% Latino (7), 15% African American/Black (6), 15% Caucasian/White (6), and 3% West Indian (1). Participants were enrolled in Introductory Psychology, Social Psychology, or Introductory Management courses and received credit toward their course research requirement.

Those in good physical and mental health as assessed by the Patient Health Questionnaire-9 (PHQ-9)^28^ were included in the study. Exclusion criteria were assessed during screening and included reports of sleep problems (e.g. insomnia or hypersomnia), circadian rhythm disorder as measured through questionnaire, use of sleeping pills, sedatives, or stimulant medications, pregnancy or possible pregnancy, dependence on nicotine or caffeine, and use of recreational drugs (e.g., marijuana, psychedelics, heroin). The protocol was approved by the Human Research Protection Program, Baruch College, City University of New York. Participants provided written informed consent prior to the medical screening and the morning assessments.

## MATERIALS

### Actigraph Watches

NES participants wore an actigraph watch (Micro Motionlogger Sleep Watch; www.ambulatory-monitoring.com) to assess sleep the night before the Final Assessments.

### Profile of Mood States (POMS-Short Form)

The Profile of Mood States-Short Form (POMS-SF)^29^ measures participants’ self-reported mood and includes a five-item fatigue subscale used in the current study.

### Morningness-Eveningness Questionnaire (MEQ)

This questionnaire^30^ was used to assess chronotype, or sleep timing preference. The total scores range from 16 (definitely evening type) to 86 (definitely morning type).

### Sleep Questionnaire

Prior to the perception of task difficulty and heuristics assessments, all potential participants were asked to report their typical nighttime sleep quality, including total sleep time, amount of sleep needed to feel refreshed, number of nightly awakenings, and insomnia symptoms (Insomnia Severity Index [ISI])^31^.

### Perception of Difficulty Assessment: Article Task and Puzzle Task

The purpose of this assessment was to determine whether total sleep deprivation affected the perception of task difficulty in comparison to those who had naturally-experienced sleep. Participants were asked for their estimates of task qualities. Such estimates could reflect a perception of greater task challenge. Participants were not asked to complete these tasks.

In the Article Task, a paper copy of an article is examined for 30 seconds. With the article in front of them, participants estimate: 1) the time they would need to read the article, 2) the number of pages the article contains (actual number = 35), 3) the number of words presented on the first page (actual number = 277), 4) how difficult it would be to read the article, and 5) how difficult it would be to write a summary of the article.

In the Puzzle Task, an unassembled jigsaw puzzle is examined for 30 seconds. With the puzzle pieces in front of them, participants estimate: 1) the time it would take them to complete the puzzle, 2) the number of pieces they believe the puzzle contains (actual number = 100), and 3) how difficult it would be to complete the puzzle.

### Heuristics Assessment: Quality Judgment Task (What-Is-Beautiful-Is-Good)

In this task, participants examine two separate images of refrigerators (previously assessed for attractiveness), each paired with a different consumer review. A photo of an attractive refrigerator is paired with an unfavorable review and a photo of an unattractive refrigerator is paired with a favorable review. Participants evaluate the refrigerator quality and report their likelihood of purchasing each refrigerator.

### Heuristics Assessment: Following Instructions Task (Greedy Algorithm)

This task has an instruction section, a reading passage, and four subsequent questions. If participants read the instructions, they know to answer only Question 4. Those who answer all four questions will have skipped the instructions.

### Heuristics Assessment: Math Difficulty-Time Choice (Speed-Accuracy Trade-Off)

In this assessment, participants expect to work on arithmetic problems for 20 minutes. After seven minutes, they are asked whether they would like to continue solving similar problems or work on more difficult problems for less time. Participants who choose the more difficult problems receive a follow-up question asking if they chose this option because they wanted to finish the task more quickly or because they desired a challenge.

## Procedure

### Recruitment

Prior to Fall 2015 (Recruitment 1), screened participants selected one of two assessment dates which were randomly determined to be NES or TSD. Students were informed of the condition after they selected a date. In Fall 2015 (Recruitment 2), immediately after screening, eligible participants were randomly assigned to NES or TSD and began their study involvement that night. During the screening, potential participants were informed of the study itinerary and continued with procedures knowing they could be randomly assigned to either sleeping at home or staying awake overnight in the lab space.

### Actigraph Pickup and Overnight Monitoring Session

NES participants began wearing the actigraph watch the day prior to the Final Assessments and slept at home that night. TSD participants arrived the night before the Final Assessments and stayed overnight at the college, monitored by research assistants. The period of extended wakefulness in TSD ranged from 22.5 to 29.5 hours, depending on individual wake time on the day prior to the Final Assessments. Neither NES nor TSD participants were permitted to consume caffeine or nicotine after 14:00 the day of the overnight session until the end of the Final Assessments, and TSD were not permitted to use electronics after 24:00 until the beginning of the assessments. The light emitted from these devices mimics sunlight, which decreases melatonin levels. Since melatonin is involved in promoting sleep, exposure to the frequency of blue light emitted by electronic devices can increase wakefulness^32^.

### Breakfast and Final Assessments

At 08:30 the following morning, the groups ate the same breakfast items; at 09:00, they began the Final Assessments (both separately).

## Design

The design of this study was between-groups with eligible participants block-randomly assigned to either the NES or TSD group. Statistical analyses were conducted comparing answers on the Final Assessments between the two groups.

## Statistical Analyses

Outcome variables with non-normally distributed data were transformed using non-linear transformations to meet the assumptions of parametric tests. Specifically, between-group effects were examined through independent groups *t* tests for continuous outcomes and chi-square analysis for binary outcomes. Outcome variables which did not meet the standard of normality, nor could be transformed to become normal, were analyzed with non-parametric Mann-Whitney *U* tests. POMS-SF fatigue served as a predictor of all outcome variables through linear regression for continuous outcomes and logistic regression for binary outcomes.

## RESULTS

### Preliminary Analyses

#### Pre-Study Sleep Characteristics

None of the assessed pre-study sleep variables differed significantly between groups. According to independent-groups *t* tests, the average nightly sleep duration for the experimental group (*M*=7.19 hrs, *SD*=0.84) was not significantly different from the control group (*M*=6.99 hrs, *SD*=1.15), *p*=.546, *t*(37) = 0.61, suggesting both groups had similar habitual sleep duration prior to the study. Other assessed sleep characteristics for each group may be viewed in Table 1.

**Table 1:** Self-reported typical sleep quality indicators in Naturally-Experienced Sleep and Total Sleep Deprivation groups.

	Naturally-Experienced Sleep	Total Sleep Deprivation	Total
	*M (SD)*	*M (SD)*	*M (SD)*
Nightly total sleep time (mins)	419.40 (50.40)	431.40 (69.00)	425.40 (60.00)
Amount of sleep needed to feel refreshed (mins)	439.80 (106.80)	398.40 (153.60)	418.20 (133.20)
Insomnia Severity Index (ISI)	5.12^a^ (3.15)	3.91^a^ (2.99)	4.50^a^ (3.09)
Number of nightly awakenings	0.50 (0.53)	0.50 (0.71)	0.50 (0.61)

Note. All data were self-reported on pre-study screening questionnaires. No significant differences were found between groups according to independent-groups t tests (not shown).

a Corresponds to no clinical insomnia.

#### Objective Measure of Sleep Quality in Naturally-Experienced Sleep Group

The total sleep time of NES ranged from 202 to 461 minutes (3.37 to 7.68 hours). The average total sleep time in NES was 5.91 hours, constituting a mild sleep deficit^33,34^. Participants slept for a significantly shorter period of time (*M*=354.74, *SD*=72.84) than they reported needing to feel rested (*M*=462.63, *SD*=84.45), *t*(18)=4.03, *p*=.001, *d*=0.93, according to paired-samples *t* tests. See Table 2 for the NES actigraph data.

**Table 2:** Objective sleep quality indicators from actigraph data in Naturally-Experienced Sleep group.

	*M (SD)*	Min	Max
Total sleep time (mins)	354.74 (72.84)	202.00	461.00
Number of awakenings per hour sleep	1.08 (0.79)	0	3.11
Duration of awakenings per hour sleep (mins)	3.62 (3.04)	0	9.56
Mean length of awakenings (entire sleep)	3.49 (2.03)	1.00	7.00
Sleep efficiency (%)	89.65% (10.56%)	63.75%	100%
Sleep onset latency (mins)	13.74 (7.40)	4.00	30.00
Sleep deficit^a^	107.89 (116.44)	-52.00	349.00
	Median	Earliest	Latest
Time fell asleep	00:17	21:59	02:35
Time woke up	06:58	05:21	09:38^b^

a Calculated as self-reported amount of sleep needed in order to feel rested minus the total sleep time recorded by the actigraph device. Negative value indicates sleep surplus; positive value indicates sleep deficit.

b One participant overslept past the designated arrival time (08:30). This participant completed the Final Assessments at 11:00 instead of 09:00 as originally intended.

#### Effects of Chronotype on Outcome Variables

The MEQ scores for NES ranged from 28 (definite evening type) to 63 (moderate morning type); the scores for TSD ranged from 38 (moderate evening type) to 62 (moderate morning type). According to independent-groups *t* tests, there were no differences between NES (*M*=51.32, *SD*=7.70) and TSD (*M*=49.00, *SD*=7.06) in chronotype as measured by the MEQ, *t*(37)=0.98, *p*=.334, *d*=0.32. On average, neither group could be classified as morning or evening type. Based on the assessment of morningness-eveningness, no participant had a circadian rhythm dysfunction. MEQ score did not significantly predict fatigue, perception of task difficulty, or use of heuristics (all *p*>.05) according to regression analyses.

#### Effects of Sleep Deprivation on Reported Fatigue

TSD reported significantly greater fatigue (*M*=18.60, *SD*=5.49) than did NES (*M*=9.37, *SD*=4.46), *t*(37)=5.92, *p*<.001, *d*=1.95, on the POMS-SF according to linear regression analyses.

#### Effects of Sleep Deprivation on Perception of Task Difficulty and Use of Heuristics

*Perception of Difficulty Assessment: Article Task and Puzzle Task*

According to independent-groups *t* tests, TSD participants estimated significantly more time would be needed to read the article (*M*=129.25, *SD*=106.16) and rated the article as significantly more difficult (*M*=4.10, *SD*=0.97) than did NES participants (estimated time: *M*=77.11, *SD*=44.64, *t*[37]=2.31, *p*=.026, *d*=0.76; difficulty rating: *M*=3.16, *SD*=1.21, *t*[37]=2.69, *p*=.011, *d*=0.88). No differences in groups were found in estimated number of pages (*p*=.737), number of words on the first page (*p*=.741), or difficulty rating for writing an article summary (*p*=.213).

**Figure 1:**
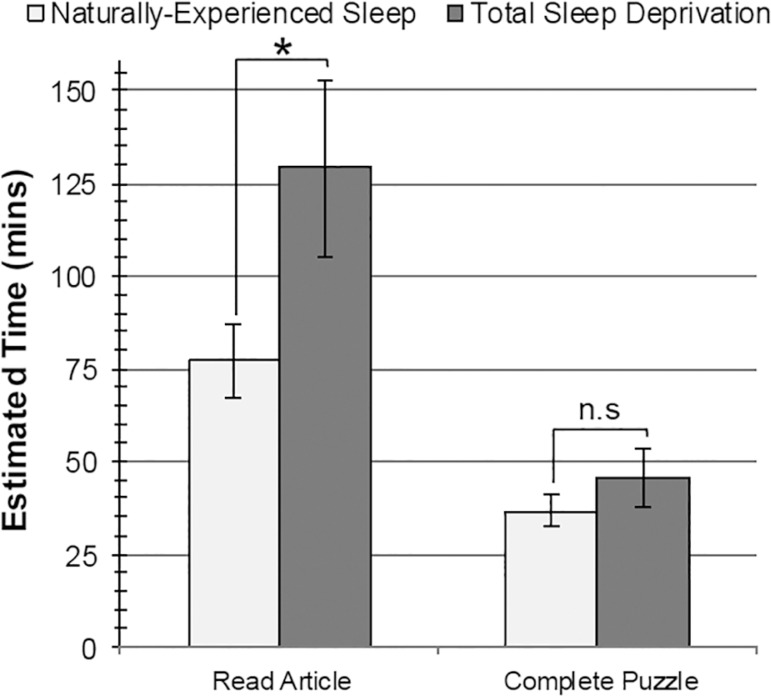
Differences between Naturally-Experienced Sleep (NES; light gray bars) and Total Sleep Deprivation (TSD; dark gray bars) on estimated time in minutes to read the article (left) and complete the puzzle (right). Error bars ± standard error of the mean. *p < .05, two-tailed. n.s. = not significant.

**Figure 2:**
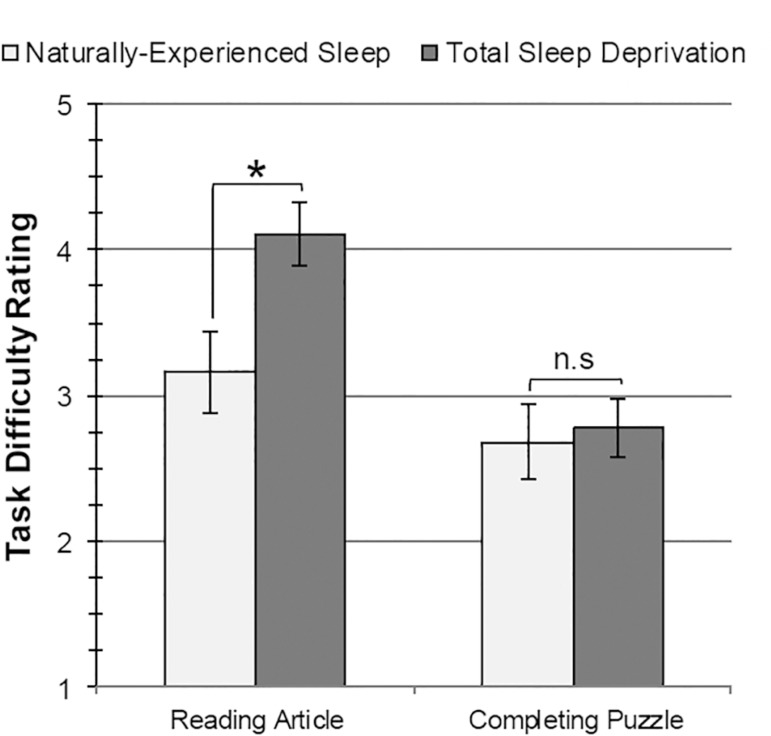
Differences between Naturally-Experienced Sleep (NES; light gray bars) and Total Sleep Deprivation (TSD; dark gray bars) on subjective task difficulty ratings (1 = Very easy, 5 = Very difficult) for reading the article (left) and completing the puzzle (right). Error bars ± standard error of the mean. *p < .05, two-tailed. n.s. = not significant.

No significant differences were found when NES and TSD groups were compared on estimated time to complete the puzzle (*p*=.511), estimated number of puzzle pieces (*p*=.142), or difficulty rating for the puzzle (*p*=.531) according to independent-groups *t* tests. See Figure 1*,* Figure 2*, and* Table 3.

**Table 3:** Differences between Naturally-Experienced Sleep and Total Sleep Deprivation in Perception of Difficulty Assessment.

	Naturally-Experienced Sleep	Total Sleep Deprivation	*t* (df)	*p*	*d*
	*M (SD)*	*M (SD)*			
Article Task					
Time (minutes)^§^	77.11 (44.64)	129.25 (106.16)	2.31 (37)	.026*	0.76
Number of pages^a§^	38.11 (21.26)	46.70 (43.72)	0.34 (37)	.737	0.11
Number of words on first page^b§^	208.68 (86.38)	309.20 (374.89)	0.33 (27.12)^c^	.741	0.13
Difficulty rating (reading)	3.16 (1.21)	4.10 (0.97)	2.69 (37)	.011*	0.88
Difficulty rating (summary)	3.53 (1.07)	3.95 (0.97)	1.27 (36)	.213	0.42
Puzzle Task					
Time (minutes)^§^	36.58 (18.19)	45.26 (34.05)	0.66 (36)	.511	0.22
Number of pieces^d§^	70.21 (26.81)	133.16 (213.76)	1.50 (36)	.142	0.50
Difficulty rating^§^	2.68 (1.11)	2.78 (0.88)	0.63 (35)	.531	0.21

*Note*. All values are estimated by the participants.

a Actual number of pages = 35.

b Actual number of words = 277.

c Levene's test for homogeneity of variance was significant (*p* < .05); t test statistic corrected through degrees of freedom was used to determine significance.

d Actual number of pieces = 100.

§ Variable has been transformed to attain normality.

* *p* < .05, two-tailed.

#### Heuristics Assessment: Quality Judgment Task (What-Is-Beautiful-Is-Good)

No significant differences between NES and TSD participants were found in reported quality rating (*p*=.163) or purchase likelihood (*p*=.223) for the attractive refrigerator according to Mann-Whitney *U* tests.

**Figure 3:**
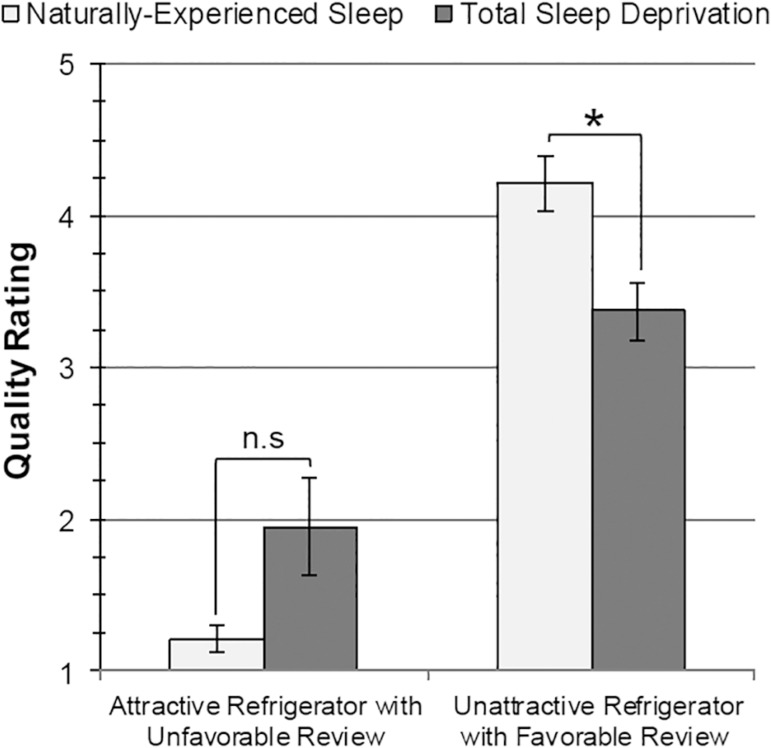
Differences between Naturally-Experienced Sleep (NES; light gray bars) and Total Sleep Deprivation (TSD; dark gray bars) in subjective quality ratings (1 = Low quality, 5 = High quality) for image of attractive refrigerator with unfavorable review (left) and unattractive refrigerator with favorable review (right). Error bars ± standard error of the mean. *p < .05, two-tailed. n.s. = not significant.

According to independent-groups *t* tests, TSD participants rated the unattractive refrigerator with the favorable review as significantly lower in quality (*M*=3.37, *SD*=0.83) than NES participants (*M*=4.21, *SD*=0.79), *t*(22.45)=-3.75, *p*=.001, *d*=-1.58. TSD participants also reported being significantly less likely to purchase this refrigerator (*M*=3.42, *SD*=1.02) than NES participants (*M*=4.00, *SD*=0.75), *t*(25.46)=-2.23, *p*=.035, *d*=-0.88. See Figure 3.

#### Heuristics Assessment: Following Instructions Task (Greedy Algorithm)

Fifty-eight percent of NES skipped instructions compared to 90% of TSD. According to chi-square analysis, TSD participants answered all four questions significantly more than NES participants, X^2^ (1)=4.89, *p*=.027. Based on the odds ratio, the odds of participants skipping the instructions were 6.18 times higher for the TSD group than for the NES group.

#### Heuristics Assessment: Math Difficulty-Time Choice (Speed-Accuracy Trade-Off)

**Figure 4:**
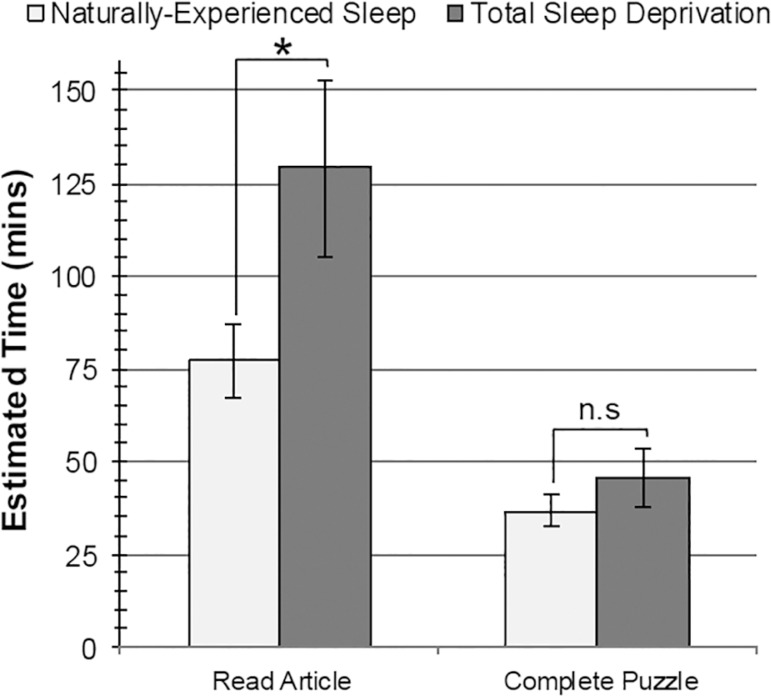
Percentage of Naturally-Experienced Sleep (NES; light gray bars) and Total Sleep Deprivation (TSD; dark gray bars) using the greedy algorithm heuristic (skipped instructions) in Following Instructions Task (left); of those choosing the more difficult math problems (n = 20; 10 each from NES and TSD), percentage of NES and TSD using the speed-accuracy trade-off (chose more difficult problems to conserve time) in Math Difficulty-Time Choice (right). ┼ *p* < .10, two-tailed. *p < .05, two-tailed.

There were no significant differences between NES and TSD groups in choice to complete more difficult math problems for less time (*p*=1.00) according to chi-square analysis. Of those who completed the challenging problems for a shorter period of time (*n*=20, 10 from each condition), a greater proportion of TSD participants chose this option to finish the task quickly (70% of TSD) compared to NES participants (30% of NES), X^2^ (1)=3.20, *p*=.074, in findings trending towards significance. TSD participants who chose the difficult math problems had 5.44 times higher odds of reporting the desire to finish the task quickly (rather than wanting a challenge) as compared with NES participants who chose the more difficult math problems. See Figure 4 and Table 4.

**Table 4:** Differences between Naturally-Experienced Sleep and Total Sleep Deprivation in Heuristics Assessment.

	Naturally-Experienced Sleep	Total Sleep Deprivation	*U*	*p*	*r*
	Median	Median			
Quality Judgment Task Attractive/ unfavorable fridge					
Quality rating^a^	1.00	1.00	132.50	.163	.28
Purchase likelihood^a^	1.00	1.00	138.50	.223	.26
	*M (SD)*	*M (SD)*	*t*(df)	*p*	*d*
Unattractive/favorable fridge					
Quality rating^§^	4.21 (0.79)	3.37 (0.83)	3.75 (22.45)^b^	.001**	-1.58
Purchase likelihood^§^	4.00 (0.75)	3.42 (1.02)	2.23 (25.46)^b^	.035*	-0.88
	%	%	X^2^		*p*
% Skipping instructions (greedy algorithm)	58%	90%	4.89		.027*
% Choosing easier math (speed-accuracy)	47%	47%	0		1.000
% Choosing difficult math to complete quickly^c^	30%	70%	3.20		.074^†^

a Analyzed with nonparametric Mann-Whitney U test.

b Levene's test for homogeneity of variance was significant (*p* < .05); t test statistic corrected through degrees of freedom was used to determine significance.

c Percentage of group reporting reasoning for choosing difficult path problems (n = 20) in order to complete task more quickly.

§ Variable has been transformed to attain normality.

† *p* < .10, two-tailed.

* *p* < .05, two-tailed.

** *p* < .01, two-tailed.

#### Reported Fatigue (POMS-SF) as Predictor of Perception of Task Difficulty and Use of Heuristics Across Conditions

#### Perception of Difficulty Assessment: Article Task and Puzzle Task

According to linear regression analyses, greater fatigue significantly predicted a higher difficulty rating for reading the article, ß=0.52, *t*(37)=3.66, *p*=.001, *R^2^* =.27, and greater estimated time to read the article, ß=0.55, *t*(37)=3.96, *p*<.001, *R^2^* =.30. In findings approaching significance, greater fatigue predicted a higher difficulty rating for writing a summary, ß=0.30, *t*(36)=1.91, *p*=.064, *R^2^* =.09. There was no association between fatigue and estimated number of pages (*p*=.127) or estimated number of words on first page (*p*=.188).

Greater fatigue significantly predicted greater estimated time to complete the puzzle, ß=0.36, *t*(36)=2.32, *p*=.026, *R^2^* =.13, according to linear regression analysis. In findings approaching significance, greater fatigue predicted estimation of a greater number of puzzle pieces, ß=0.30, *t*(36)=1.87, *p*=.070, *R^2^* =.09. Fatigue levels were not significantly associated with the difficulty rating for the puzzle (*p*=.164).

#### Heuristics Assessment: Quality Judgment Task (What-Is-Beautiful-Is-Good)

According to linear regression analyses, greater fatigue significantly predicted a higher quality rating for the attractive refrigerator with the unfavorable review, ß=0.45, *t*(36)=3.06, *p*=.004, *R*=.21, and predicted a greater likelihood of purchasing this refrigerator in findings approaching significance, ß=0.30, *t*(36)=1.92, *p*=.063, *R^2^* =.09.

Greater fatigue significantly predicted a rating of lower quality for the unattractive refrigerator with the favorable review, ß=-0.34, *t*(36)=-2.16, *p*=.038, *R^2^* =.12, according to linear regression analysis; however, there was no association between reported fatigue and purchase likelihood for this refrigerator, *p*=.205.

#### Heuristics Assessment: Following Instructions Task (Greedy Algorithm)

No significant association between reported fatigue and skipping the instructions for the Following Instructions Task was found (*p*=.204) according to logistic regression analysis.

#### Heuristics Assessment: Math Difficulty-Time Choice (Speed-Accuracy Trade-Off)

A logistic regression showed greater fatigue predicted choice of easier math problems in findings approaching significance, X^2^ (1)=3.29, *p*=.070, *R_N_^2^ =* .11. Among those choosing the more difficult math problems, no relationship between reported fatigue and reasoning for choosing these problems was found (*p*=.122).

## DISCUSSION

### Overview and Implications of Findings

#### Sleep Deprivation and Perception of Task Difficulty

Sleep-deprived participants expected that reading the article would be more difficult and that more time would be necessary to complete the task. The assessment of the specific, countable aspects of the task, including number of pages and number of words, was unaffected by sleep deprivation. These findings suggest that while perception of objective task elements is unchanged after total sleep deprivation, sleep loss results in expected performance limitations and a decrease in estimation of one›s own ability.

If individuals perceive tasks as more difficult following sleep deprivation or insufficient sleep, they are reflecting the impaired status of the system, and they may be less motivated to expend effort because the task appears to be-and perhaps is-less feasible. The increased perception of difficulty for the Article Task may result in the reduction in motivation to complete such a task. One study, for example, found that self-reported motivation decreased progressively throughout completion of a task perceived as difficult^35^. It was hypothesized that this reduced engagement was due, in part, to progressively decreasing expectations of successful task completion.

According to Bandura›s Self-Efficacy Theory^36^, an individual’s level of perceived self-efficacy determines the extent of effort they will expend in the face of adversity and the length of time they will persist at obstacles. Expectations of greater self-efficacy will lead to more intense efforts when facing difficult tasks. Thus, individuals who experience total sleep deprivation and perceive tasks to be more difficult may also perceive themselves to be less able to perform these tasks, creating a self-fulfilling prophecy. These findings have implications in settings such as school and the workplace, where individuals’ assessment of their own ability to perform tasks is likely to impact initial engagement and performance outcomes.

#### Sleep Deprivation and the Use of Heuristics

The tendency to perceive physically attractive stimuli as possessing favorable traits is known as the what-is-beautiful-is-good heuristic^21^. Sleep-deprived participants gave a lower quality rating and were less likely to purchase the unattractive refrigerator than the participants who had slept. Those who had not slept therefore used the what-is-beautiful-is-good heuristic to a greater extent than did the controls. Greater reported fatigue predicted a higher quality rating for the attractive refrigerator and a rating of lower quality for the unattractive refrigerator. The use of the what-is-beautiful-is-good heuristic in the sleep-deprived participants may be explained by the elevated fatigue levels, or the use of this heuristic may reflect a common underlying physiological response to sleep deprivation.

The what-is-beautiful-is-good stereotype has been explored in a study of cognitive load^37^. When cognitive load was high, consumer products which were unattractive but paired with superior consumer reviews were deemed as low quality (i.e., judged through their negative physical appearance). When cognitive load was low, participants judged these products as higher quality (i.e., judged based on the favorable consumer review). Similarly, the participants in the present study likely experienced a limitation on cognitive resources and greater cognitive load due to sleep deprivation^38^ and judged the unattractive refrigerator with the favorable consumer review by its negative appearance rather than its favorable review. These findings are of considerable importance since sleep deprivation may result in heuristic processing and judgment based on appearance rather than the systematic, effortful processing of the important details of a stimulus. Such limitations in processing may influence judgment in critical situations in the workplace and personal settings. Future research may determine whether sleep deprivation affects stereotypic judgment of gender, age, and ethnicity.

Sleep-deprived participants used the greedy algorithm heuristic^22^ during the Following Instructions Task. Instead of reading the instructions, the sleep-deprived participants completed more questions on the task than needed and thus spent more time than required. Total sleep deprivation seems to limit the thorough examination of stimuli and instead promotes decision making which relies on automatic behavior. Such skipping of instructions saves energy and time in the short term but can lead to errors, especially when instructions provide unique information.

In assessment of the speed-accuracy trade-off^23^, among participants who chose the more difficult math problems (10 participants in each condition), sleep-deprived participants had over five times the odds of choosing these problems in order to complete the task more quickly when compared with participants who slept at home. These findings trended toward significance, likely due to the smaller sample of participants (*n*=20) choosing the more difficult problems who answered the follow-up question. Such findings corroborate that total sleep deprivation imposes limitations on effort. In comparison to the control group, sleep-deprived participants preferred to limit the time engaged on the task, utilizing a time-conservation strategy. This strategy appears to be an attempt to exert effort for less time, indicating they “traded” short-term cognitive resources for escape from the task.

Though heuristics are used frequently in everyday life and can often help individuals, they can also have deleterious consequences. In medical residents, who often suffer from a lack of sleep, those with more experience were found to use the availability bias heuristic; they made decisions in new cases based on previous cases rather than using analytical reasoning^39^. Similarly, overconfident venture capitalists used heuristics by making decisions based on past successes without taking time to process new information that would improve their accuracy, resulting in more incorrect decisions^40^. Overall, the findings from the current study indicate the importance of sleep for engagement in systematic mental processes.

### The Role of Fatigue in Perception of Task Difficulty and Use of Heuristics

In the current study, sleep deprivation induced greater fatigue and predicted a higher difficulty rating for reading the article, a greater estimated amount of time to read the article, greater estimated time to complete the puzzle, a higher quality rating for the attractive refrigerator, and a rating of lower quality for the unattractive refrigerator. Though sleep-deprived participants, compared to those who slept, were less likely to purchase the unattractive refrigerator with the favorable review, skipped instructions, and trended toward choosing difficult math problems to save time, fatigue was not associated with any of these outcomes. The considerable variability in sensitivity to the effects of subjective fatigue on cognitive performance may explain these findings^41^. That is, sleep may lead to a limitation in cognitive resources^38^ which is accompanied by greater subjective fatigue in some individuals, but not in others. Future studies may identify predictors of sensitivity to subjective fatigue following sleep deprivation, and which factors predict inter-individual differences in the association between fatigue and use of cognitive heuristics.

The findings from the current study indicate that fatigue induced by sleep deprivation may influence critical decision making outside of the lab environment. Judges who are sleep deprived and use the what-is-beautiful-is-good heuristic may make more favorable rulings for attractive people. Sleep-deprived healthcare professionals may ignore hand-washing instructions, and physicians who have not obtained sufficient sleep may wish to complete tasks quickly, such as prescribing antibiotics rather than discussing alternative treatment plans. Sleep-deprived law enforcement may be more likely to judge a criminal suspect as a threat based on the suspect›s fulfillment of stereotypes, critically impacting the officer›s decision to act with force against the perceived threat.

Sleep loss increases fatigue and affects millions. Approximately 29% of adults in the United States report getting less sleep than they need each night, with 27% of respondents reporting being unable to work efficiently because they are too sleepy^42^. Given the findings from the current study, millions of American adults may be vulnerable to perception of greater difficulty and use of heuristics and, consequently, errors in judgment and decision making. This is particularly concerning given the necessity of making decisions based on the careful estimation of the alternatives and logic rather than expedience. The present findings therefore emphasize the importance of sleep in reducing perceptions of task difficulty, successful completion of such tasks through utilization of effortful mental processes, and preservation of decision-making skills.

### Future Directions

Patterns of adenosinergic activity in the nucleus accumbens (NAcc) may constitute the physiological substrates of behavioral effort reduction induced by sleep loss. Neural activity during wake coincides with elevated metabolism and increased concentration of extracellular adenosine in the central nervous system^43^. During sleep, cortical interstitial space increases dramatically, allowing for the removal of toxins^44^ including adenosine, higher levels of which are correlated with the subjective experience of fatigue^45^. Sleep deprivation results in the up-regulation of adenosine receptors^46^ in or close to the NAcc shell^47^. Adenosine, acting on A_2_A receptors in opposition to the dopamine (DA) D_2_ receptor system, modulates the activity of GABAergic neurons within the NAcc, reducing arousal and initiating sleep via multiple inhibitory projections throughout the arousal system^47^. It is the A_2_A receptors, for example, that are uniquely receptive to the arousal effects of caffeine^48^.

Separate from sleep investigations, researchers examining effort-related decision making have found that the receptors responsible for arousal inhibition and sleep promotion also regulate behavioral effort. Adenosine regulates effort-related processes through a selective interaction between adenosine A_2_A receptors and antagonists of DA D_2_ receptors^49^. For example, A_2_A antagonists can reverse the behavioral effects of DA antagonists on effort-related choice behavior^50^. These researchers suggest that stress on this system may be responsible for fatigue and psychomotor slowing^51^. Sleep deprivation may, indeed, be a stressor affecting the interaction of A_2_A and D_2_ receptor systems. Sleep is promoted through adenosinergic activity via the A_2_A receptors and effortful behavior is inhibited at the same synapses. Future studies examining the production of adenosine during wakefulness, stimulated up-regulation of adenosine receptors in the NAcc after sleep deprivation, and adenosinergic projections that inhibit arousal may help clarify the mechanisms in the cascade responsible for reduced behavioral effort due to sleep loss.

### Limitations

This was a naturalistic study; the purpose was to compare individuals maintaining their typical sleep patterns with those who were totally sleep deprived. The participants were randomly assigned to experience either total sleep deprivation or sleep at home according to their natural sleep patterns. Thus, in the home sleep group, we did not control the amount of sleep obtained. As a result, some participants who slept at home had less sleep on the night prior to the Final Assessments than they reported needing in order to feel fully rested, as indicated by the actigraph data. In essence, the control group was given the opportunity for full sleep but instead experienced a naturally-induced sleep deficit.

Physiological and cognitive changes consistent with some sleep loss, including fatigue, are likely to have been experienced in this group. In the current study, increased levels of fatigue were predictive of effort-related performance impairments, with the greatest effort-related impairments produced following no sleep. The inadequate amount of sleep experienced by the control group is consistent with other studies which have found that less than one third of college students receive eight hours or more of sleep each night^52^.

Thus, the average sleep length for the control group may offer a realistic representation of college students’ sleep habits. Nonetheless, our results suggest that even under natural sleep conditions, when participants are permitted to sleep as they would normally, increased fatigue is related to greater perceptions of task difficulty and use of heuristics on effort-related tasks. Thus, total sleep loss impairs effort-related performance when compared with a naturally-experienced minor sleep deficit. Our findings also suggest that having some sleep confers benefits on effort-related performance in comparison to the total absence of sleep. Future experimental studies designed to enforce adequate sleep will clarify differences in effort between the full complement of sleep and naturally-experienced sleep, which may include naturally-experienced sleep loss.

The absence of between-group differences on some variables could be explained by the partial sleep loss experienced by those who slept at home. Specifically, perception of difficulty in the Puzzle Task, quality rating and purchase likelihood for the attractive refrigerator with the unfavorable review, and the Math Difficulty-Time Choice did not significantly differ between groups. Future studies which compare the use of heuristics by those who have had their full sleep complement with those who have been sleep-deprived might show greater between-groups differences in these variables.

Participants in NES and TSD experienced different settings for the overnight session on the night prior to the Final Assessments; the former slept in their home environment, and the latter remained awake in the sleep laboratory. This experimental design allowed for the examination of NES participants› performance following a night in their natural sleep environment as opposed to the unfamiliar lab setting. Sleeping in an unfamiliar environment may result in poorer sleep quality or quantity, also known as the first night effect^53^. Having participants sleep at home was intended to reduce the first night effect.

However, exposure to different settings prior to the Final Assessments could affect performance in subtle ways. The effects, if any, of differences in pre-assessment context on perception of task difficulty and use of heuristics could be assessed in future studies. Furthermore, participants’ sleep duration and quality were not assessed in the period prior to the overnight monitoring session; thus, we cannot conclude that both groups had similar sleep patterns immediately prior to entering the study. The groups, however, did not differ on any of the self-reported variables measured through the sleep questionnaire.

Moreover, participants were randomly assigned to groups which mitigated any potential sleep differences between the groups. In addition, though participants were randomly assigned to conditions, future studies would benefit from a within-subjects assessment to provide an unequivocal understanding of the impact of no sleep on the use of heuristics. The available tasks used to assess perception of difficulty and use of heuristics do not have equivalent parallel forms, which precluded a within-subjects design in the current study.

## CONCLUSION

Our findings demonstrate the effects of sleep deprivation and fatigue on perception of task difficulty and use of heuristics. Sleep deprivation induces greater self-reported fatigue, which is associated with perception of greater task difficulty. Due to this change in perception, sleep-deprived individuals may attempt to compensate for their limitations by using heuristics rather than complex mental processes. In the current study, the sleep-deprived participants perceived the Article Task as more difficult and used the what-is-beautiful-is-good, greedy algorithm, and speed-accuracy trade-off heuristics. The results from this study emphasize the importance of examining the various ways in which sleep deprivation and fatigue affect the perceived difficulty of tasks, effort expenditure, and critical real-world outcomes.
